# Effect of a Cognitive Function and Social Skills-Based Digital Exercise Therapy Using IoT on Motor Coordination in Children with Intellectual and Developmental Disability

**DOI:** 10.3390/ijerph192416499

**Published:** 2022-12-08

**Authors:** Seung-Bo Park, Yumi Ju, Hyunjin Kwon, Heeok Youm, Min Joo Kim, Jinwook Chung

**Affiliations:** 1 Department of Sports Science Convergence, Dongguk University, 30 Pildong-ro 1-gil, Jung-gu, Seoul 04620, Republic of Korea; 2 Human Development and Rehabilitation, Graduate School of Education Service Science, Dongguk University, 30 Pildong-ro 1-gil, Jung-gu, Seoul 04620, Republic of Korea; 3 Department of Sport Culture, Dongguk University, 30 Pildong-ro 1-gil, Jung-gu, Seoul 04620, Republic of Korea; 4 Department of Mechanical Engineering, Kyung Hee University, Yongin-si 17104, Republic of Korea

**Keywords:** internet of things (IoT), virtual reality exercise system, cognitive function, social skills, intellectual disability, developmental disability

## Abstract

This study aimed to determine the effects of a virtual reality exercise program based on cognitive function and social skills on motor coordination in children with intellectual and developmental disabilities (IDD). Thirty-five children with IDD were randomly assigned to either the cognitive function and social skills-based virtual reality exercise system (CS-VR) group or the conventional virtual reality exercise system (C-VR) group. Before and after the intervention, each participant was tested for motor coordination (extended horizontal jump, hop, stationary dribble, overarm throw) and exercise performance (standing long jump, YMCA step test). Compared with the C-VR group, the CS-VR group showed significant improvements in motor coordination in terms of extended horizontal jump, hop, and overarm throw (*p* < 0.01, *p* < 0.05, and *p* < 0.01, respectively). In addition, compared with the C-VR group, the CS-VR group showed a significant increase in standing long jump (*p* < 0.01), although no significant between-group variation was found in stationary dribble and recovery heart rate (RHR) as part of the YMCA step test (*p* > 0.05, and *p* > 0.05, respectively). These results suggest that for the development of motor skills in children with IDD, it is essential to develop an exercise program that reflects the levels of cognitive functions and social skills of these children.

## 1. Introduction

Children with intellectual and developmental disabilities (IDD) exhibit a state of physical, learning, linguistic, or behavioral disability due to their respective impairments. The associated symptoms generally start during the period of growth and persist throughout their lifetime [1]. Individuals with IDD include those with general mental impairment that affects adaptive function in three domains: conceptual, social, and practical skills [2]. Specifically, as reported by the American Association on Intellectual and Developmental Disabilities (AAIDD), IDD is a functional limitation in learning, reasoning, problem-solving, and adaptive behaviors related to conceptual, social, and practical skills [3,4]. Although modern medicine does not offer treatment for IDD, physical activity may improve the quality of life of individuals with IDD and the quality of motor coordination, gait, mobility, and performance of daily activities [5].

Several studies have reported that children with IDD exhibit relatively low levels of strength, muscular endurance, flexibility, motor coordination, and cardiorespiratory endurance than those without IDD [6]. The reported causes include a sedentary lifestyle, low levels of motivation or intellectual ability, short attention span, limitations and disabilities related to motor development, lack of motivation to commit to the test, and other psychological and physiological factors [7]. To resolve such problems, guiding children to continuously engage in exercise is essential, especially regarding the improvement of health and physical fitness [8]. Consequently, for individuals with IDD, developing an exercise program that reflects the cognitive, social, and emotional development of the patient, as well as incorporates the areas and levels of their personal interests, is recommended [9].

Internet of Things (IoT)-based applications that collect exercise data can motivate students to exercise more by allowing them to quickly track their activity status and compare it with their own objectives [10]. The IoT is a system of interconnected computing devices or things that are equipped with sensors and have the capacity to transport data across a network and interact intelligently with one another [11]. In this regard, recent methods to encourage physical activity in patients with IDD apply information and communications technology. Among such interventions, a well-known approach is virtual reality (VR) [12]. By offering a virtual world that resembles the actual environment, VR has been shown to have positive effects in improving cognitive and social skills in adolescents with IDD [13]. Previous studies suggest that the utilization of VR technology may provide an effective platform to increase the level of physical activity through immersion and motivation [12]. Despite such benefits, it remains questionable whether children with IDD who exhibit varying levels of cognitive function and social skills can successfully perform VR exercise programs.

The close association between physical activity and executive functions, such as cognitive flexibility and planning, has been reported to pose challenges to the performance of physical activity by children with IDD in response to complex external stimuli [14]. In addition, social dysfunction may reduce participation in physical activity and opportunities of interaction [15], which makes it difficult to maximize the effects of VR exercise programs. This implies that the positive effects of VR exercise programs in improving motor coordination can be maximized if the difficulty of the program can be adjusted according to the levels of cognitive function and social skills in children with IDD.

However, no study has investigated VR exercise programs that consider the cognitive functions and social skills of children with IDD. This study aimed to determine the effects of a cognitive function and social skills-based VR exercise program on motor coordination in children with IDD. We hypothesized that children with IDD participating in a VR exercise program based on cognitive function and social skills will show improved motor coordination and physical abilities compared to a conventional VR exercise program.

## 2. Materials and Methods

### 2.1. Subjects

Thirty-five children (aged 8–13) with mild IDD participated in this study. Children from public and special schools, as well as local welfare centers, were recruited as participants through internet advertisements. The participants were children who lacked experience related to adapted physical education outside regular school curriculum and who did not perform regular exercise. The parents or guardians of the children provided written informed consent after understanding the purpose and methodology of the research. This study was approved by the institutional ethics committee of Dongguk University (DUIRB-202204-11). The protocols applied in this study complied with the Declaration of Helsinki. The inclusion criteria in this study were as follows: (1) sedentary lifestyle (no participation in regular exercise over the past year); (2) ability to interact with others in linguistic and non-linguistic ways; (3) no history of cardiovascular, respiratory, and musculoskeletal disorders, and medication-free (all candidates had a medical interview and a 12-lead resting ECG examination to confirm eligibility by a doctor). The exclusion criteria were: (1) physical disability or sensory disturbance (visual or auditory impairment) and (2) neurological disorders (e.g., vestibular disorder, multiple sclerosis, Parkinson’s disease, peripheral neuropathy, head injury, stroke).

### 2.2. Study Design

**Figure 1:**
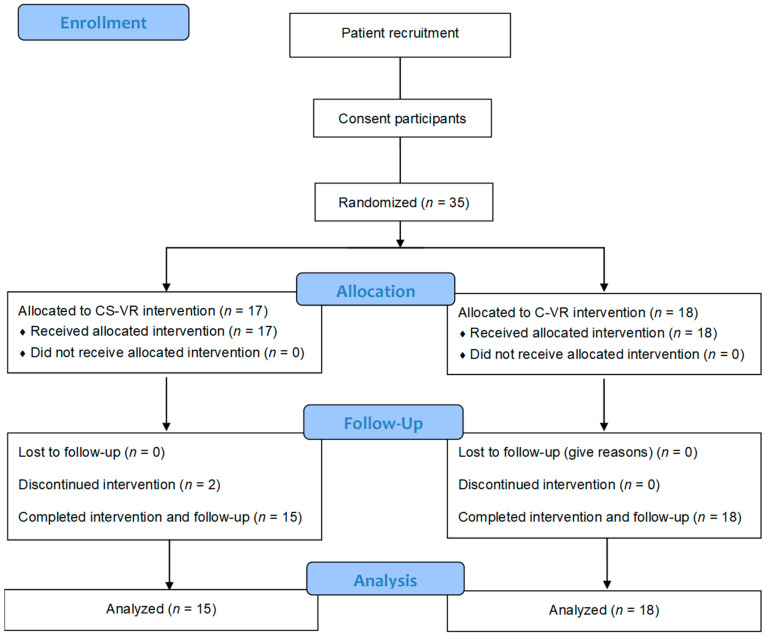
CONSORT flow diagram of methodology and data analysis.

Thirty-five children with IDD were randomly assigned to the following two groups: the cognitive function and social skills-based virtual reality exercise system (CS-VR) or the conventional virtual reality exercise system (C-VR). Randomization was stratified by age, sex, and diagnosis. Two subjects in the CS-VR group were excluded due to three or more absences from the CS-VR intervention. Finally, of the 33 subjects, 15 in the CS-VR group (12 boys and 3 girls) and 18 in the C-VR group (14 boys and 4 girls) completed each intervention program (Figure 1). For 2 days, the participants underwent evaluations of cognitive functions and social skills, as well as the Test of Gross Motor Development-2 (TGMD-2) to test exercise performance. On the first day of the visit to the laboratory, anthropometric parameters of the participants were measured, while they were given explanations of the overall experimental protocol and risk factors. All participants underwent two sessions to familiarize themselves with the VR exercise program. The CS-VR group underwent a VR exercise program based on cognitive function and social skills scores. The IoT-based CS-VR was interconnected with a tablet-based application that collects cognitive functions and social skills data. The participants were tested before and after an 8-week CS-VR or C-VR intervention. Both the groups underwent a VR exercise program comprising whole-body exercises once a week.

### 2.3. Motor Coordination and Exercise Performance Test

Motor coordination and exercise performance tests in children with IDD were evaluated with reference to the literature on physical fitness and motor development [16,17,18,19,20]. Several discussions were held regarding findings from the literature and the evaluation items among an expert group that included one research professor each from the field of motor development, adapted physical education, exercise physiology as well as a pediatric psychiatrist. Consequently, the items were drawn for the Physical Activity Promotion System for Students with Disabilities (PAPS-D), which is a physical fitness evaluation tool developed in South Korea for children with developmental disabilities [20] as well as for TGMD-2, which is a motor development evaluation tool that can be used for children with developmental disabilities [21]. The individuals who provided the TGMD-2 rating included a research professor, an expert in motor development who had conducted a study on the reliability and validity of TGMD-2 for use in South Korea [22], and one PhD researcher with an acceptable level of inter-rater reliability. They followed the conventional methods suggested in the TGMD-2 guidelines, while the inter-rater reliability was verified based on Cronbach’s α (Cronbach’s α = 0.91).

To evaluate gross motor development in children, the TGMD-2 tests six items for locomotion skills and six items for object control skills to provide information on the level of development in the same age group. A set of selected items was applied in this study, considering the distinct characteristics of children with developmental disabilities (extended horizontal jump, hop, stationary dribble, over arm throw). The total score is based on the sum of the raw scores obtained by repeating each item twice. Assuming that all items were successfully performed by the participant, the sum of raw scores for each of the three to five items is distributed in the range of 6–10.

#### 2.3.1. Extended Horizontal Jump

The extended horizontal jump was evaluated as per the performance criteria of standing long jump for assessment of exercise performance. The measurements were performed in duplicated, and each scene was recorded using a video camera positioned to one side. The recording was subsequently evaluated based on the criteria listed in Table 1. for the rating [22]. The total score was based on the sum of the raw scores obtained by repeating each item twice.

#### 2.3.2. Hop

The participant stands on one leg and hops forward after being instructed to jump from the starting line as far as possible, once with the left foot and then with the right foot. The measurements were performed in duplicate, and each scene was recorded using a video camera positioned to one side. The recording was subsequently evaluated by two motor development experts based on the criteria listed in Table 2 [22]. The total score was based on the sum of the raw scores obtained by repeating each item twice.

#### 2.3.3. Stationary Drills

The total score was based on the sum of the raw scores obtained by repeating each item twice. Stationary dribbling was performed by the participants, and they were instructed not to move their feet and use only one hand to bounce the ball four times or more before catching it. The measurements were performed in duplicate, and each scene was recorded using a video camera positioned to one side. The recording was subsequently evaluated by two motor development experts based on the criteria listed in Table 3 [22]. The total score was based on the sum of the raw scores obtained by repeating each item twice.

#### 2.3.4. Overarm Throw

An overarm throw was performed by the participants, whereby they were instructed to stand on a marked line 6.1 m away from a wall and throw the ball towards it with maximum force. The measurements were performed in duplicate, and each scene was recorded using a video camera positioned on one side. The recording was subsequently evaluated by two motor development experts based on the criteria listed in Table 4 [22]. The total score was based on the sum of the raw scores obtained by repeating each item twice.

#### 2.3.5. Standing Long Jump

In this study, we tested the standing long jump of individuals using the markerless motion capture technology Kinect (Microsoft, Redmond, WA, USA) and the active remote sensing system Intel Lidar (Intel RealSense, Santa Clara, CA, USA). Azure Kinect and Intel Lidar together made up the Kinect assessment system, and their purpose was to collect precise data regarding the participant’s movement performance. After a single demonstration, the participant was given time to practice two to three times if necessary. The measurements were performed in duplicate. The distance from the starting line to the heel closest to the starting line was recorded.

#### 2.3.6. YMCA Step Test

A step box with a height of 20 cm was used, and a step test was conducted in accordance with an established protocol [23]. Each participant stepped up and down 24 times per minute for 3 min in synchronization with a metronome set at 96 beats per minute. At the end of the test, the participant sat on a chair nearby, and the recovery heart rate (RHR) for one minute (Polar H10, Polar Electro OY, Kempele, Finland) was immediately measured.

### 2.4. Cognitive Function Test

**Figure 2:**
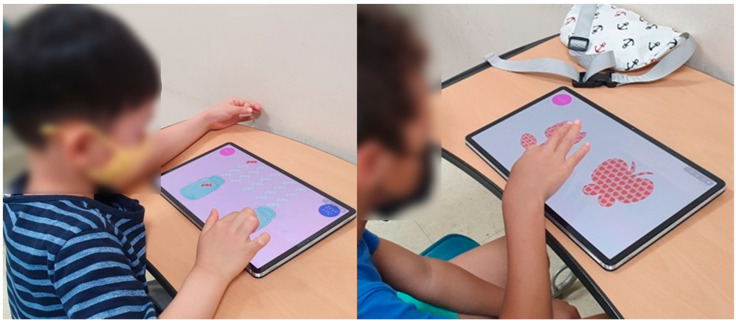
Digital cognitive function testing using a tablet-based app, patent application 10-2020-0147889 (Republic of Korea, 2022).

To assess cognitive functions in children with developmental disabilities, a tablet-based app was used (Figure 2) [24]. The assessment consisted of eight items as follows: spatial working memory, working memory span, visual working memory, global and local visual processing (congruent vs. incongruent), and executive function (planning, response inhibition, inhibition, and attention). The maximum score of each sub-item was 10 for a total score of 80. The time taken for the assessment was approximately 20 min, with variations according to the level of cognitive function across participants. The details of the sub-items are listed in Table 5. A receiver operating characteristic (ROC) analysis was performed to determine the cutoff score for the upper and lower ranges of cognitive function. For a total score of ≥55.5, the participants were classified into a higher-level CS-VR exercise program. For a total score of <55.5, the participants were classified into the lower-level CS-VR exercise program.

### 2.5. Social Skills Test

**Figure 3:**
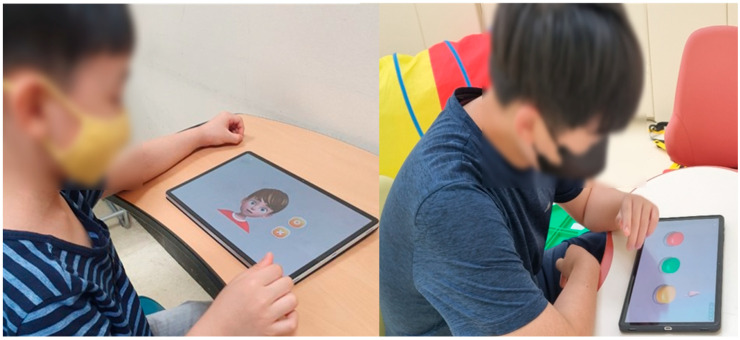
Digital social skills testing using a tablet-based app, patent application 10-2020-0168431 (Republic of Korea, 2022).

A tablet-based app was used to assess the social skills of children with developmental disabilities (Figure 3) [24]. The assessment consisted of eight items as follows: imitation, joint attention, pre-symbolic knowledge, symbolic behaviors, understanding rules, emotion perception, and perspective-taking. The maximum score of each sub-item was 10 for a total score of 80. The time taken for the assessment was approximately 20 min, with variations according to the level of social skills across the participants. The details of the sub-items are listed in Table 6. A ROC analysis was performed to determine the cutoff score for the upper and lower ranges of social skills. For a total score of ≥64.5, the participants were classified into a higher-level CS-VR exercise program. For a total score of <64.5, the participants were classified into the lower-level CS-VR exercise program.

### 2.6. VR Room Setup

**Figure 4:**
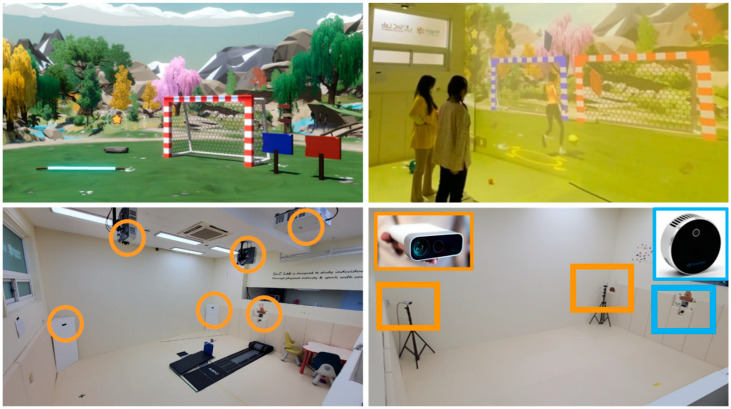
IoT-based virtual reality exercise system.

A VR exercise program was conducted in a classroom-sized environment of 5 m × 5 m × 4 m, with a screen spanning 5 m × 4 m. In addition, to construct visual and audio simulations within the space, virtual reality projectors, Kinect, Lidar, speakers, personal computers, and monitors were employed (Figure 4). The IoT-based CS-VR group was provided with tablet-based app information that interconnected cognitive functions and social skills data of the individual levels, but not the VR group.

### 2.7. VR Exercise Program

**Figure 5:**
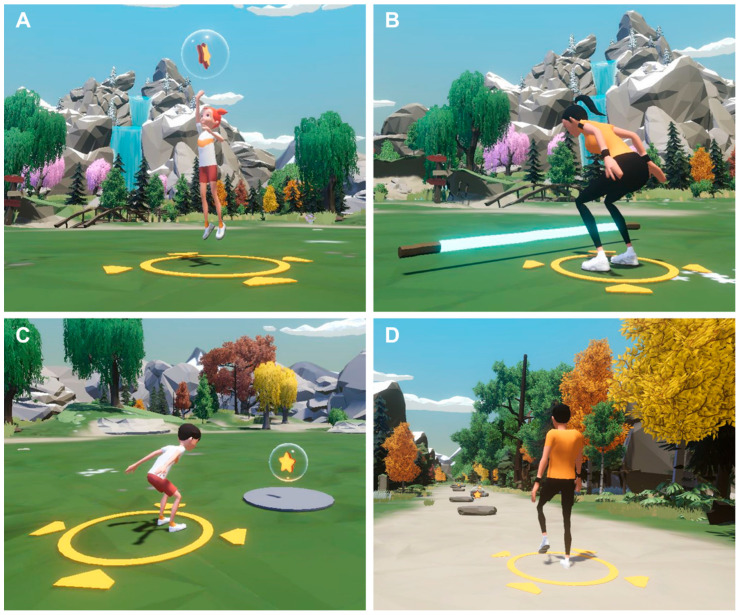
(**A**) Illustration of the vertical jump, (**B**) Illustration of the hurdle jump, (**C**) Illustration of the horizontal jump, (**D**) Illustration of the walking exercise, Patent 10-2427137 (Republic of Korea, 2022).

The VR exercise program consisted of one psychiatrist, two exercise physiologists, one expert in adapted physical education, and four graduate teaching assistants majoring in sports psychology. Once per week, 60 min sessions of intervention with VR exercise were scheduled as follows: 10 min for dynamic stretching, 40 min for exercise, and 10 min for feedback and cooling down (Table 7). The VR exercise program consisted of a horizontal jump, running, high jump, walking exercise, bar hurdle, step exercise, ball-kick, ball throw, balance exercise, and animal-like motion (Figure 5).

The differences in the level of difficulty of the following 10 exercises were as follows: (1) horizontal jump (increase in number of circles with an increase in difficulty); (2) running (running on one specified path to a path diverged to two paths); (3) high jump (increase in the jump height and pop bubbles); (4) walking exercise (increase from one path to two paths to search for fruit-shaped items); (5) bar hurdle (jumping over a stationary hurdle to jumping over consecutive approaching hurdles); (6) step exercise (popping bubbles on an approaching step box and an increase in difficulty of popping bubbles that are randomly placed on right or left step boxes); (7) ball-kick (Increase from one goal to two goals and kicking according to the mission); (8) ball throw (throwing a ball by choosing the big picture of butterfly or flower and choosing the big picture of butterfly or flower over the small picture of butterfly or flower); (9) balance exercise (increase in difficulty from balancing on a balance beam to balancing on a balance beam while performing activities); (10) animal-like motion (from simply imitating the actions of animals shown on the screen to remembering the order and imitating the actions of animals shown after the screen is off). The details of the VR intervention programs are listed in Table 8.

For CS-VR and C-VR intervention, maintaining a target heart rate of 60% HRmax intensity was recommended [25,26]. The average heart rate was recorded before and after each exercise. Each participant performed an exercise in accordance with the level of difficulty, based on his or her cognitive and social skills. After 4 weeks, the level of difficulty increased according to individual proficiency.

### 2.8. Statistical Analysis

All statistical analyses were performed using SPSS version 25.0 (IBM corp., Armonk, NY, USA). Parameters are presented as mean ± SD. The assumption of normality was assessed using the Shapiro–Wilk test, and sphericity was examined using Mauchley’s test. Changes in motor coordination and exercise performance factors were analyzed using repeated measures analysis of variance (RM-ANOVA) with interventions (VR exercise program and control group) and time (baseline and post-intervention) as repeated variables with two levels each. The effect size (ES) was determined using partial eta-squared (ηp2) from RM-ANOVA results, with an effect size of small (≥0.0099 and <0.0588), moderate (≥0.0588 and <0.1379), and large (≥0.1379) effects, respectively [27]. Post-hoc tests for changes after interventions in the VR intervention or control group were compared using a paired *t*-test. The statistical significance level was set at *p* < 0.05. The ES was computed by Cohen’s d value and thresholds for small, medium, and large effects were <0.5, 0.5–1.2, and >1.2, respectively [28].

## 3. Results

### 3.1. Participants’ Demographics

The participants’ anthropometric parameters, including mean ± standard deviation (SD), were as follows: age 10.7 ± 1.5 years, height 139.7 ± 13.7 cm, and weight 41.2 ± 12.3 kg. The participants’ clinical and demographic characteristics are summarized in Table 9.

### 3.2. Extended Horizontal Jump

**Figure 6:**
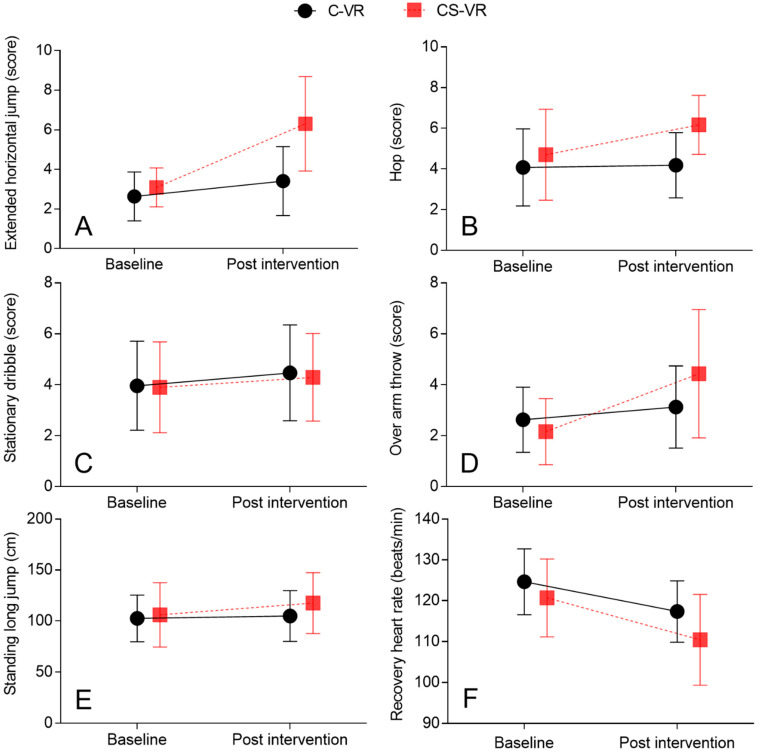
(**A**) Comparison of the mean of extended horizontal jump score between CS-VR and C-VR, F_(1, 31)_ = 12.452, *p* < 0.01, (**B**). Comparison of the mean of hop score between CS-VR and C-VR, F_(1, 31)_ = 6.705, *p* < 0.05, (**C**). Comparison of the mean of stationary dribble score between CS-VR and C-VR, F_(1, 31)_ = 0.084, *p* = 0.773, (**D**). Comparison of the mean of over arm throw score between CS-VR and C-VR, F_(1, 31)_ = 7.654, *p* < 0.01, (**E**). Comparison of the mean of standing long jump between CS-VR and C-VR, F_(1, 31)_ = 11.01, *p* < 0.001, (**F**). Comparison of the mean of RHR between CS-VR and C-VR, F_(1, 31)_ = 5.432, *p* < 0.001. Error bars indicate standard deviation (SD).

A significant difference was noted in the change in extended horizontal jump score between the CS-VR and C-VR groups (interaction F_(1, 31)_ = 12.452, *p* < 0.01; intervention F_(1, 31)_ = 7.447, *p* < 0.05; time F_(1, 31)_ = 33.58, *p* < 0.001) (Figure 6) (Table 10). Further, in response to the CS-VR intervention, the extended horizontal jump score changed significantly (t_(14)_ = 4.824, *p* < 0.001, d = 3.247, time). In response to C-VR intervention, there was a significant change in extended horizontal jump score (t_(17)_ = 2.613, *p* < 0.05, d = 0.629, time).

### 3.3. Hop

A significant difference was noted in the change in hop score between the CS-VR and C-VR groups (interaction F_(1, 31)_ = 6.705, *p* < 0.05; intervention F_(1, 31)_ = 2.419, *p* = 0.13; time F_(1, 31)_ = 9.083, *p* < 0.01) (Figure 6) (Table 10). Moreover, in response to the CS-VR intervention, the hop score changed significantly (t_(14)_ = 2.899, *p* < 0.05, d = 0.656, time). However, the hop score did not alter significantly in response to the C-VR intervention (t_(17)_ = 0.489, *p* = 0.63, d = 0.059, time).

### 3.4. Stationary Dribble

No significant differences were noted in the stationary dribble scores between the CS-VR and C-VR groups (interaction F_(1, 31)_ = 0.084, *p* = 0.773; intervention F_(1, 31)_ = 0.738, *p* = 0.396; time F_(1, 31)_ = 6.825, *p* < 0.05) (Figure 6) (Table 10). Further, in response to CS-VR intervention, there were no significant differences in the stationary dribble scores (t_(14)_ = 1.702, *p* = 0.11, d = 0.225, time). Furthermore, the hop score did not alter significantly in response to the C-VR intervention (t_(17)_ = 2.034, *p* = 0.057, d = 0.286, time).

### 3.5. Overarm Throw

A significant difference was noted in the change in the overarm throw score between the CS-VR and C-VR groups (interaction F_(1, 31)_ = 7.654, *p* < 0.01; intervention F_(1, 31)_ = 0.001, *p* = 0.974; time F_(1, 31)_ = 18.77, *p* < 0.001) (Figure 6) (Table 10). Moreover, in the CS-VR group, the overarm throw score changed significantly (t_(14)_ = 3.445, *p* < 0.01, d = 1.741, time). In response to C-VR intervention, there was a significant change in the overarm throw score (t_(17)_ = 2.474, *p* < 0.01, d = 0.39, time).

### 3.6. Standing Long Jump

A significant difference was noted in the change in the standing long jump between the CS-VR and C-VR groups (interaction F_(1, 31)_ = 11.01, *p* < 0.01; intervention F_(1, 31)_ = 0.667, *p* = 0.42; time F_(1, 31)_ = 24.9, *p* < 0.001) (Figure 6) (Table 10). In response to the CS-VR intervention, the standing long jump changed significantly (t_(14)_ = 5.432, *p* < 0.001, d = 0.367, time). In the CS-VR group, the standing long jump increased by 11.01% compared with that at baseline; however, the standing long jump did not alter significantly in the C-VR group (t_(17)_ = 1.278, *p* = 0.218, d = 0.102, time).

### 3.7. Recovery Heart Rate (RHR)

No significant differences were noted in RHR between the CS-VR and C-VR groups (interaction F_(1, 31)_ = 0.965, *p* = 0.333; intervention F_(1, 31)_ = 4.426, *p* < 0.05; time F_(1, 31)_ = 33.27, *p* < 0.05) (Figure 6) (Table 10). However, in response to CS-VR intervention, a significant change was noted in RHR (t_(14)_ = 3.673, *p* < 0.01, d = 1.076, time). Further, in response to the C-VR intervention, RHR changed significantly (t_(17)_ = 5.432, *p* < 0.001, d = 0.367, time).

## 4. Discussion

Our results showed that compared with the VR group, the CS-VR intervention group showed significant improvements in motor coordination (extended horizontal jump, hop, and overarm throw) after the intervention of the cognitive functions and social skill-based VR exercise program. The results suggest that the intervention tailored to the individual levels of cognitive functions and social skills of children with IDD before the exercise program had positive effects in terms of improving motor coordination for motions with varying levels of complexity.

In general, the standing long jump is a representative method to assess the maximum muscular power (high-speed strength) of the lower limbs at maximum strength, with explosive force in a short period of time as the muscles contract [29]. For the standing long jump, biomechanical factors such as the maximum shoulder flexion angle, the shoulder joint angle upon takeoff, and the size of the moment generated in the lower limbs have been reported as additional critical factors [30]. Similarly, for hops, coordination between the hip and knee joints to readily allow takeoff and landing as well as the rhythm and timing of the arms in forward motion and the legs to generate momentum is important [31]. Hence, the standing long jump (high-speed strength), extended horizontal jump, and hop assessed in this study are closely associated with motor coordination, dynamic balance, attentional control, control, and postural control systems, and these kinematic parameters may be particularly crucial for children with IDD [32]. As a result of the positive effects of the CS-VR exercise system, long jump, high jump, stepping, and bar hurdles showed improvements in motor coordination, including visual-motor coordination (the coordination between the limbs and the visual stimuli). These findings suggest that improvements in visual-motor coordination and motor coordination (motor performance) may be maximized by providing a tailored exercise intervention that reflects the levels of cognitive function and social skills of each individual, as movements such as long jumps and hops with relatively high levels of difficulty require the individual to learn the basic patterns and slowly get used to the motions.

In this study, the performance of overarm throws was significantly higher in the CS-VR intervention group than that in the VR group. With regards to the throwing motions, the overarm throw was the most complex exercise, whereby the acceleration at all segments of the body occurred in coordinated series (right foot, left foot, trunk, throwing arm, throwing hand), which is the most important determinant of the maximum absolute velocity at the hand [33]. This indicates that the ball throw (throwing the ball at a flying butterfly) and ball-kick (kicking the ball through the goal post) sections of the CS-VR exercise program developed in this study can be important variables in the improvement of object control skills. These results are also presumed to be indicative of improved motor coordination, including visual-motor coordination [34]. Raygoza-Romero et al. [35] reported that an exergame that combines exercise and a VR-enhanced game with a visual factor could improve visual-motor coordination in children with a spectrum of neurodevelopmental disorders, and serve as a potential tool to support visual-motor coordination. Milajerdi et al. [36] reported that an exergame intervention could improve motor skills and executive functions in 60 children aged 6–10 years with autism spectrum disorders, which was in agreement with the results of this study. Edwards et al. [37] and Kohl et al. [38] reported that the positive effects on object control skills were attributable to the transition of control exercises to game and sports, as social interactions led to a sport-like activity. However, previous studies report that the positive effects were from a simple VR game that did not consider each individual’s cognitive function and sociality; therefore, additional research is warranted to improve object control skills based on the results of our study.

A close bidirectional relationship exists between physical activity and motor skills (motor performance), and physical activity has been demonstrated as a positive influencing factor for motor development in children with IDD [39]. In a study by Ku [40], the total movement skill scores were significantly higher for moderate-to-high-intensity physical activity than that for low-intensity activity in preschool-aged children. In this study, no significant difference was noted in cardiorespiratory endurance between the interventions, with both the groups showing significant improvement over time. However, this indicates the crucial impact of significantly improved cardiorespiratory endurance on motor coordination in the CS-VR group of children with IDD. It seems that the improvement in cardiorespiratory endurance in the CS-VR intervention and the combined effect of motor intervention suitable for each individual’s cognitive function and sociality level had an essential effect on improving motor coordination in children with IDD. Consequently, the CS-VR and C-VR program in this study allowed the average heart rate (beats/min) to be maintained at a moderate level or above, which is in line with previous studies. To maximize improvements in motor coordination, the level of exercise for children with IDD should be set to a moderate level or above.

Meanwhile, no significant difference was observed for stationary dribble between the CS-VR and C-VR. A common factor among effective exercise interventions is that exercise movements in these interventions coincide with target movements [40]. However, movements in the intervention in this study did not coincide with stationary dribbles as an object control skill. Sit et al. [41] targeted improvements in kicking, throwing, running, jumping, and grip in children with IDD, and the exercise program with a focus on the five exercise techniques was shown to have produced positive effects on fundamental movement skills. In a similar study conducted on 66 children with IDD, an aerobic running training group showed improvements in body mass index, sit-ups, and cardiorespiratory fitness, whereas a Tai Chi training group showed improvements in lower limb muscle strength and coordination [42]. Fragala-Pinkham et al. (2008) reported that an aquatic aerobic exercise program (twice a week for 14 weeks) led to improvements in cardiopulmonary fitness and running performance in 16 children with IDD, aged 6–11 years (11 males and 5 females), although no significant variation was found for muscle strength or motor skill (motor performance) [43]. Previous studies collectively suggested that to enhance the effects of an exercise program for children with IDD, an intervention targeting motor skills is recommended [40,41,43], which was in line with the results of this study.

Some limitations should be considered when interpreting the results and implications of the present study. In this study, the level of exercise was classified based on cognitive and social function evaluation results, and progression i.e., an increase in exercise intensity around the time the participants adapted to the exercise, was applied after 4 weeks of intervention, but the respective level could not be clearly quantified. Nevertheless, for each of the ten exercise types, the level of difficulty was increased if the three experts (one psychiatrist, one exercise physiologist, and one expert in adapted physical education) determined that the participant showed a certain level of proficiency.

## 5. Conclusions

The cognitive functions and social skills-based VR exercise program developed in this study was shown to have positive effects on improving motor coordination and exercise performance in children with IDD. The results highlight the importance of developing an exercise intervention that reflects the levels of cognitive and social skills in children with IDD for motor development. Future studies should develop disability and function-specific exercise programs as interventions based on VR technology.

## 6. Patents

In parallel to this paper was developed the patent number (10-2427137, Republic of Korea, 2022).

## Figures and Tables

**Table 1:** Extended horizontal jump.

Performance Criteria
1. An initial pose is formed with arms stretched behind and knees bent.
2. Both arms are thrust upwards with force towards the top of the head.
3. Both feet completed a simultaneous jump before touching the ground together.
4. Both arms are stretched downwards during landing.

The maximum total score was eight, with a maximum score of four for each measurement.

**Table 2:** Hop.

Performance Criteria
1. The leg without load swings like a pendulum to create force.
2. The leg without load is positioned on the side of the body.
3. Arms are bent and in a swing to create force.
4. The dominant or preferred leg is used to perform three or more consecutive leaps and landings.
5. The non-dominant or non-preferred leg is used to perform three or more consecutive leaps and landings.

The maximum total score is 10 with the maximum score of 5 at each measurement.

**Table 3:** Stationary drills.

Performance Criteria
1. One hand comes into contact with the ball at the height of the navel.
2. The ball is pushed by the fingers and not the palm.
3. Ball contacts surface in front of or to the outside of foot on the preferred side.
4. The feet are not to be moved during four consecutive dribbles.

The maximum total score was eight, with a maximum score of four for each measurement.

**Table 4:** Overarm throw.

Performance Criteria
1. Windup is initiated with downward movement of hand/arm.
2. The ball is pushed by the fingers and not the palm.
3. Weight is transferred by stepping with the foot opposite the throwing hand.

The maximum total score was eight, with a maximum score of four for each measurement.

**Table 5:** Cognitive function assessment.

Sub-Item		Screenshot	Task Description
Working Memory	Spatial Working Memory	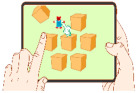	Find the teddy bear in the box after showing and covering it
	Working Memory Span	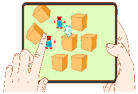	Find multiple (2–7) teddy bears in order
	Visual Working Memory	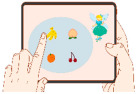	Find the changed fruit in four locations
Global and Local Processing of Visual Perception	Congruent	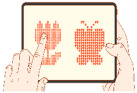	Find the congruent flower and butterfly according to voice instructions
	Incongruent	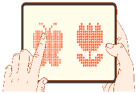	Find the incongruent flower and butterfly according to voice instructions
Executive Function	Planning	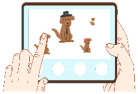	Place the figures such as dog, cat, bone etc. in order by size
	Response Inhibition	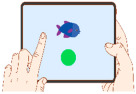	Press the button when a fish appears, hold the button when a shark appears
	Inhibition and Attention	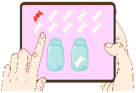	Press the glass bottle without candy while not pressing the other bottle with candy

The maximum score of each sub-item was 10 for a total score of 80.

**Table 6:** Social skills assessment.

Sub-Item		Screenshot	Task Description
Activity Participation	Following Instructions	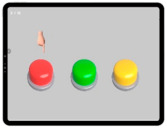	Follow the instructions and press the button that the finger pointed to
	Imitation	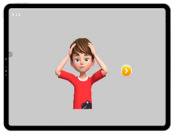	Imitate the posture or movement of the avatar
Solitary Play	Joint Attention	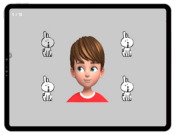	Touch the object where the gaze of the avatar’s eyes is directed
	Pre-symbolic knowledge	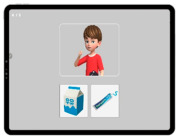	Select the correct tool to use for the avatar’s behavior
	Symbolic behaviors	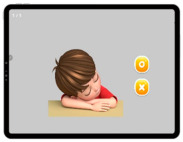	Imitate the behavior according to the verbal instruction using the avatar’s cue
Cooperative Play	Understanding rules	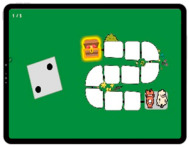	Play the dice game with an examiner by taking turns
	Emotion Perception	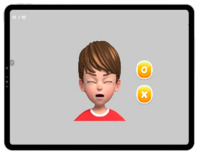	Recognize facial and emotional expressions
	Perspective taking	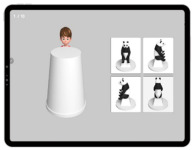	Select the view of the panda from the avatar’s perspective

The maximum score of each sub-item was 10 for a total score of 80.

**Table 7:** VR exercise program.

Section	Time	Exercise	Frequency
Warm up	10 min	Dynamic stretching	1 session/ week
Main part (VR exercise)	40 min	Horizontal jump Running High jump Walking exercise Bar hurdle Step exercise Ball-kick Ball throw Balance exercise Animal-like motion	
Cool down	10 min	Feedback and Dynamic stretching	

Recommended to maintain a target heart rate of 60% HRmax intensity during VR exercise.

**Table 8:** Descriptions of VR exercise program for each level.

Sub-Item		Details
Horizontal Jump	Low-level	- In addition to the existing circle, another circle is created in front of the teacher and the child at a 30 cm distance. Upon the ‘Jump’ signal, the teacher jumps into the circle to stimulate simulation in the child. - If the child jumps successfully, the music and lighting become more captivating. Special flashing and lighting effects and additional sounds are played to stimulate the child’s interest. - If the child exits the circle, the special effects and sounds are stopped to prevent them from leaving the circle.
	High-level	- A circle containing items (e.g., star balloon) is created. - Two circles, with and without items, respectively, are created simultaneously. - A circle with items and two without are created simultaneously. - A circle with items and up to four without items are created simultaneously. - The child must jump into the circle with items to succeed.
Running	Low-level	- Five bubbles are created in random locations. - The teacher demonstrates popping all the bubbles and instructs the child to follow. - When the child pops the bubbles, they obtain the items included within.
	High-level	- When the child pops the bubbles, they obtain the items within. - When a bubble is popped, the next bubble is created in a random location. - The bubbles must be popped within 15 s before they disappear.
High Jump	Low-Level	- A bubble is shown on the screen at a height of 10 cm from the child pelvis. - The teacher demonstrates jumping and popping the bubble. - When the child jumps and pops the bubble, the item within is obtained.
	High-Level	- The child pops four bubbles after watching the teacher’s demonstration. The height of the bubbles gradually increases (10, 20, 30, and 40 cm from the pelvis). - The child obtains the items in the bubbles after popping them.
Walking Exercise	Low-Level	- Three different paths are shown to the child: straight line, circle, and zigzag. - Bubbles with fruits inside are placed at various intervals along the path. - When the child steps on the bubbles, they pop, releasing the items.
	High-Level	- A Y-shaped path is shown on the screen. - At the end of the left and right paths, bubbles are created, containing different types of fruit. - A bubble with a specific fruit can be selected to walk in the corresponding direction. - After the item is obtained, a new Y-shaped path is created. - The child must successfully obtain a total of 10 items.
Bar Hurdle	Low-Level	- Various bars are created on the floor view. - The teacher demonstrates jumping over the bars in the front view. - The child jumps over the bars on the floor upon the audible ‘Jump’ signal.
	High-Level	- Three bars approach the child consecutively on the floor view. - The child jumps over the bars on the floor upon the audible’ Jump’ signal.
Step Exercise	Low-Level	- Step boxes appear on the path. As the boxes approach them, the child performs one step exercise and then resumes walking. - Bubbles with items are placed on the step boxes. The bubbles pop as the child steps over the boxes.
	High-Level	- Bubbles with and without items are randomly placed on the left or right step boxes. - The child only steps over the bubbles with items to perform the exercise successfully. - When the child steps over the boxes, the bubbles pop, and the items are obtained.
Ball Kick	Low-Level	- A goalpost is placed in the center of the front screen. - When the teacher avatar raises a flag above its head, the child kicks the ball towards the goalpost. - The child succeeds if they score a goal.
	High-Level	- Two goalposts in blue and red, respectively, are placed on the left and right sides of the front screen. - When the teacher raises a blue or red flag, the child kicks the ball towards the goalpost of the corresponding color. - The child succeeds if they score a goal.
Ball Throw	Low-Level	- A butterfly and a flower are displayed on the left and right sides of the front screen. - Following the teacher’s demonstration, the child is instructed to throw the ball toward either the butterfly or the flower.
	High-Level	- Small butterflies are grouped to create a large flower shape. - Small flowers are grouped to create a large butterfly shape. - The child is instructed to throw the ball toward a specific large shape, regardless of the small shapes. - The child succeeds upon hitting the correct large shape.
Balance Exercise	Low-Level	- In the front view, the teacher demonstrates walking on a balance beam and instructs the child to walk on the beam. - Sounds effects are played when the child is not on the beam to induce them to stay on it.
	High-Level	- The teacher demonstrates walking on the balance beam in the front view and instructs the child to repeat the movement. - A part of the balance beam is highlighted in a square, and the child performs activities within that beam portion. - The child performs three different movements while balancing on the beam.
Animal-Like Motion	Low-Level	- The teacher shows tiger/dog/cat movements on the screen. - The child is instructed to memorize and imitate the movements performed by the teacher. - The teacher visually ensures that the imitations are correct.
	High-Level	- The teacher’s avatar imitates three animals, and then the screen is turned off. The child is instructed to imitate the three animals in the right order. - The teacher visually ensures that the imitations are correct.

A 40-min exercise program included 10 exercises.

**Table 9:** Clinical and demographic characteristics of the participants.

P#	CS-VR (*n* = 15)	Age	Sex	P#	C-VR (*n* = 18)	Age	Sex
P1	ASD	10	M	P1	ASD	13	F
P2	ASD	13	M	P2	ASD	9	M
P1	MID	10	F	P3	ASD	11	M
P4	ASD	9	M	P4	MID	9	M
P5	MID	12	M	P5	ASD	12	F
P6	DS	10	F	P6	MID	8	M
P7	ASD	10	M	P7	DS	8	M
P8	ASD	11	M	P8	ASD	12	M
P9	MID	12	M	P9	MID	12	M
P10	MID	11	F	P10	ASD	11	F
P11	ASD	8	M	P11	MID	12	M
P12	ASD	13	M	P12	ASD	13	M
P13	ASD	10	M	P13	DS	11	F
P14	ASD	9	M	P14	ASD	12	M
P15	MID	9	M	P15	ASD	11	M
				P16	MID	10	M
				P17	MID	11	M
				P18	ASD	10	M

P#: participants number, M: male, F: female, CS-VR: cognitive function and social skills-based virtual reality exercise system, C-VR: virtual reality exercise system, ASD: autism spectrum disorder, MID: mild intellectual disability, DS: down syndrome.

**Table 10:** Changes of performance variables in response to CS-VR and VR (mean ± SD).

Variable	CS-VR			C-VR			Time × Intervention Interaction	Effect Size (η_p_^2^)
	Pre Mean (SD)	Post Mean (SD)	MD	Pre Mean (SD)	Post Mean (SD)	MD		
Extended horizontal jump (score)	2.4 ± 1.41	5.6 ± 2.38	3.2	2.33 ± 1.23	3.11 ± 1.74	0.78	*p* = 0.001	0.287
Hop (score)	4 ± 2.23	5.46 ± 1.45	1.46	3.77 ± 1.89	3.88 ± 1.6	0.11	*p* = 0.015	0.178
Stationary dribble (score)	3.2 ± 1.78	3.6 ± 1.72	0.4	3.66 ± 1.74	4.16 ± 1.88	0.5	*p* = 0.773	0.003
Over arm throw (score)	1.46 ± 1.3	3.73 ± 2.52	2.27	2.33 ± 1.28	2.83 ± 1.61	0.5	*p* = 0.009	0.198
Standing long jump (cm)	105.33 ± 31.63	116.93 ± 29.91	11.6	102.27 ± 22.91	104.61 ± 24.77	2.34	*p* = 0.002	0.316
Recovery heart rate (beats/min)	120 ± 9.53	109.73 ± 11.06	−11	124.33 ± 8.1	117.05 ± 9.08	−7.28	*p* = 0.333	0.023

Data are presented as mean ± SD of *n* = 33, MD: mean difference, SD: standard deviation.

## Data Availability

Data are available on request due to restrictions e.g., privacy or ethical. The data presented in this study are available on request from the corresponding author. The data are not publicly available due to the privacy of Dongguk University.
